# Supplementary figures and images for: Zebrafish optic nerve injury results in systemic retinal ganglion cell dedifferentiation
Source: PLoS Genet. 2025 Sep 19;21(9):e1011879. doi: 10.1371/journal.pgen.1011879 (PMC12459811; doi:10.1371/journal.pgen.1011879)

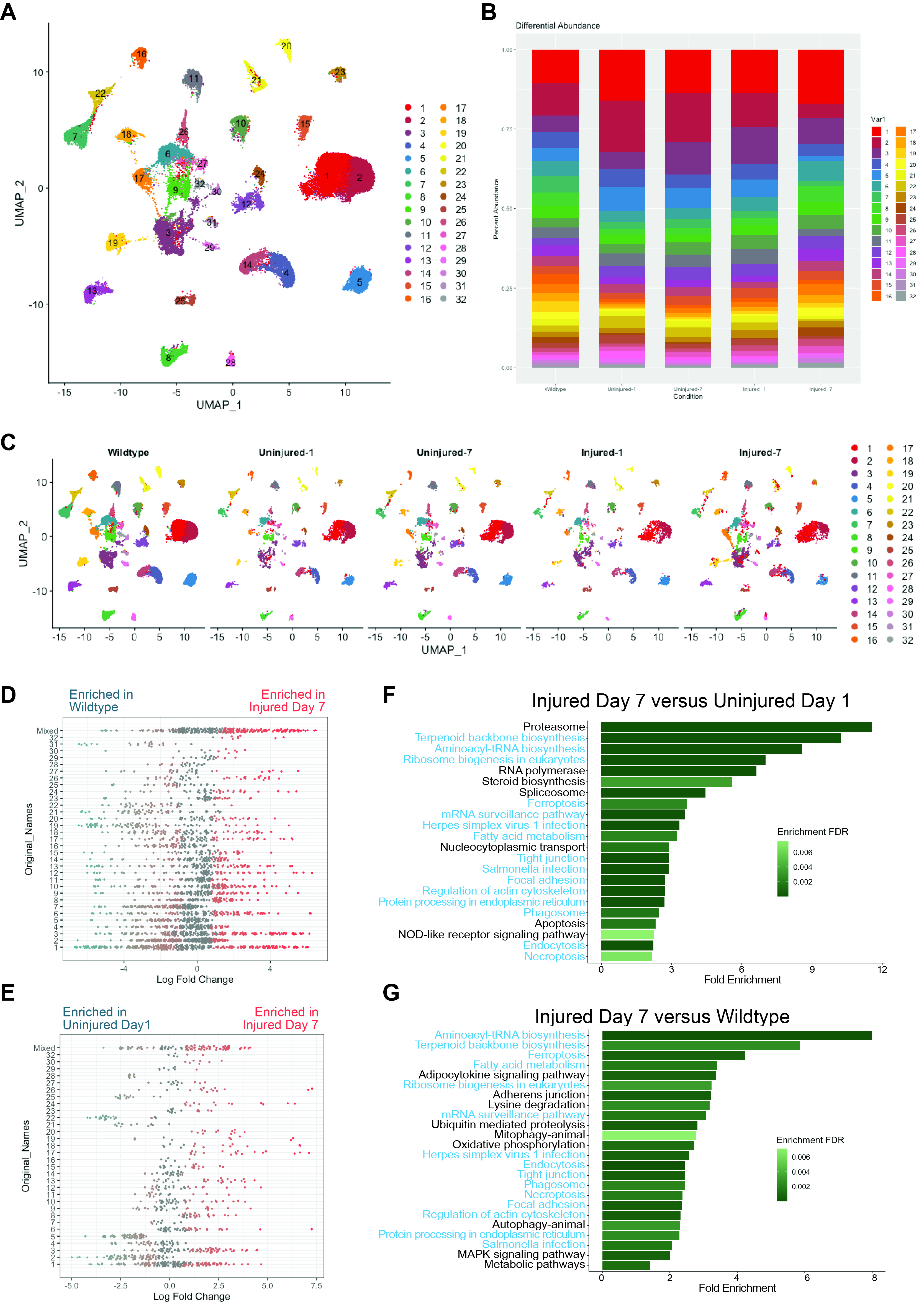

Supplement: S1 Fig — We performed a parallel analysis to assess our data relative to a previously published adult zebrafish wildtype RGC single cell dataset [25]. We found that an integrated cluster analysis showed representation of each of the five datasets (Wildtype, Uninjured Day 1, Uninjured Day 7, Injured Day 1 and Injured Day 7) in all clusters. Although we detect an uninjured eye response in our Uninjured Day 7 dataset (Fig 7), we found that our Uninjured Day 1 dataset showed consistent similarities to the wildtype, uninjured dataset [25]. We therefore chose to use our Uninjured Day 1 dataset as our control comparison. By only using data generated in the current study, we avoided some batch effects in our experiment relative to the previously published data. This figure shows the similarities between previously published wildtype data and the Uninjured Day 1 data. A) Uniform manifold approximation and projection (UMAP) of all data generated in this study integrated with the Kölsch et al., 2021 dataset [25]. This independent analysis identified 32 clusters [25]. B) Differential abundance bar graph of each subtype across the datasets, including the Kölsch et al., 2021 dataset labeled as wildtype. C) The integrated UMAP of data generated in this study and previously published wildtype adult data, split by each dataset: Wildtype [25], Uninjured Day 1, Uninjured Day 7, Injured Day 1 and Injured Day 7. D,E) Milo plots for differential abundance comparison. Milo analysis shows differential occupancy of cells in KNN graphs. This is a Beehive plot showing the differential abundance of each subtype. Each dot is a neighborhood and the dots toward the right (red) are neighborhoods enriched in the Injured Day 7 dataset. The dots toward the left are enriched in the Wildtype or Uninjured Day 1 dataset respectively. The assigned cluster number is on the left. Mixed, means the neighborhood occupies two clusters D) Comparison of wildtype versus Injured Day 7 and E) Comparison of Uninjured Day 1 ve [file pgen.1011879.s005.tif]

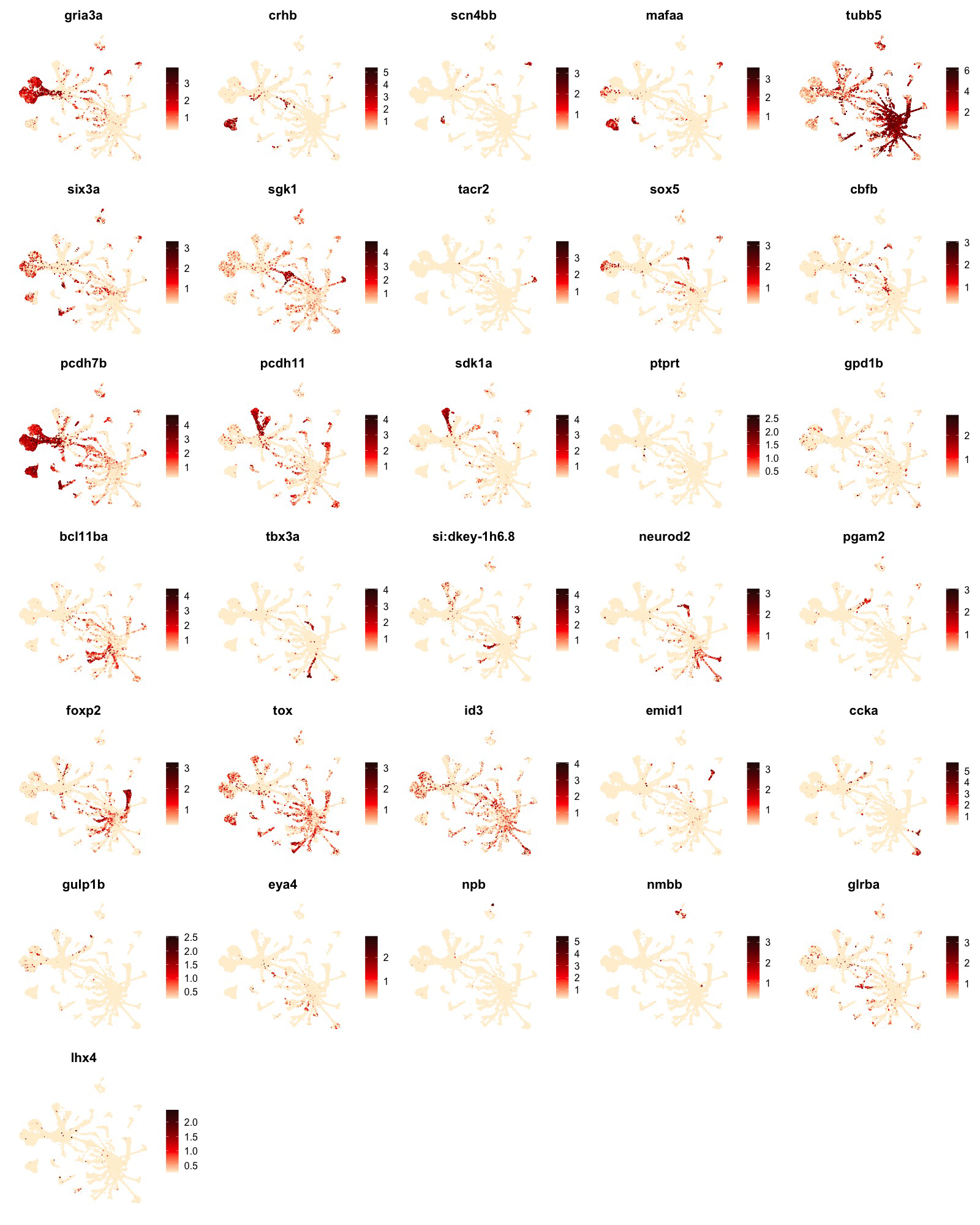

Supplement: S2 Fig — A) Percent mitochondrial genes per cell separated by replicate and organized by sample showing low percentage of mitochondrial genes overall. B) UMAP representation of each replicate, organized by experimental condition showing consistency across replicates C) UMAP representation of each experimental condition grouped by replicate. This representation shows that each cluster includes cells from each replicate. D) UMAP representation of all merged uninjured data, both Uninjured Day 1 and Uninjured Day 7. 31 clusters were found. The colors do not correlate with our final integrated analysis shown in Fig 1. E) UMAP representation of all injured data, both Injured Day 1 and Injured Day 7. 24 clusters were found. The colors do not correlate with the final integrated analysis shown in Fig 1. E and 1F are a result of a merged, not integrated, pipeline. F) Violin plot of isl2b expression in pseudobulk analysis of Uninjured Day 1, Uninjured Day 7, Injured Day 1 and Injured Day 7 RGCs showing downregulation after injury. (TIF) [file pgen.1011879.s006.tif]

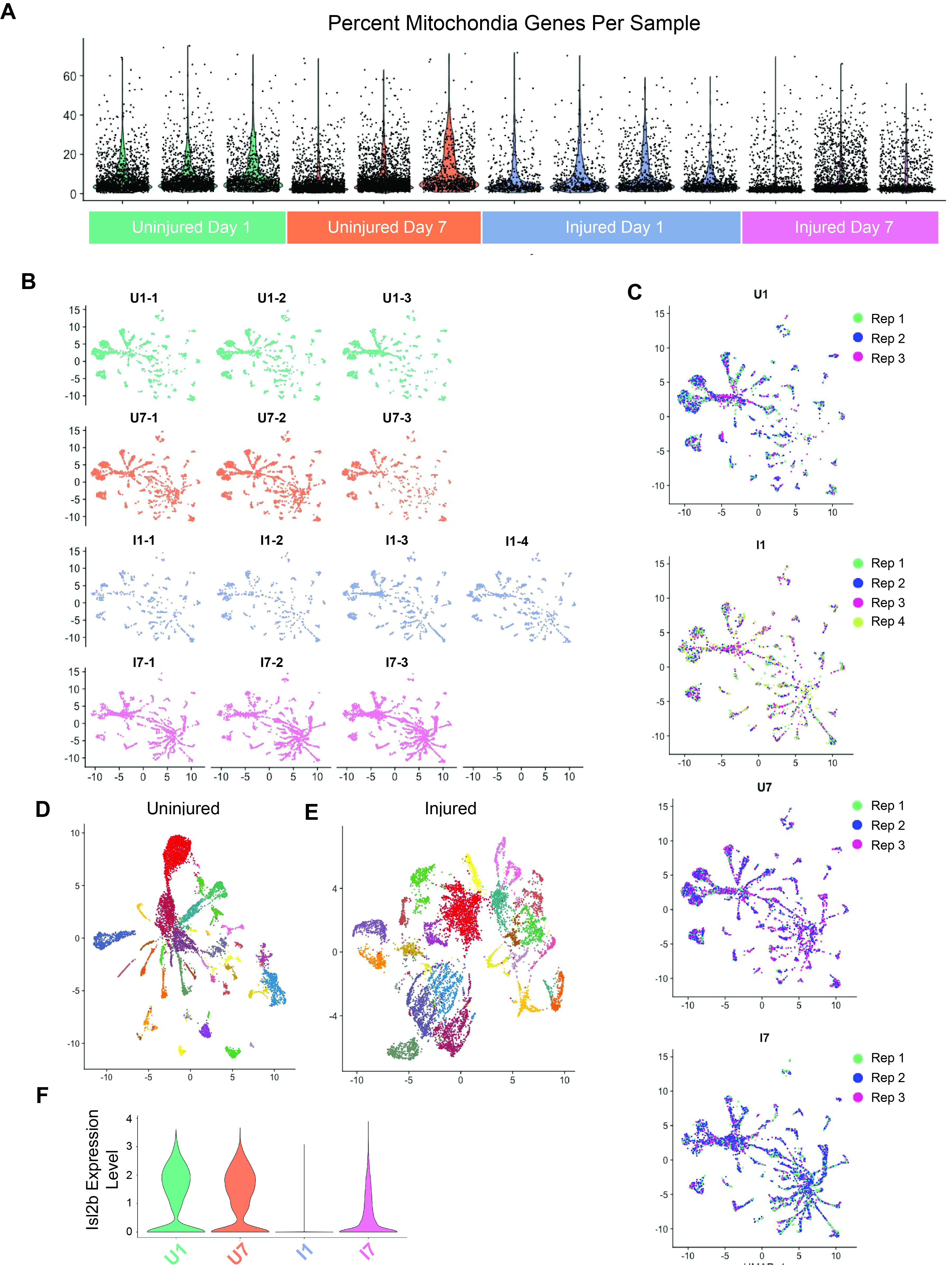

Supplement: S3 Fig — Corresponding feature plots with a min.cuttoff of q25 for the RGC subtype markers shown in Fig 1D. (TIF) [file pgen.1011879.s007.tif]

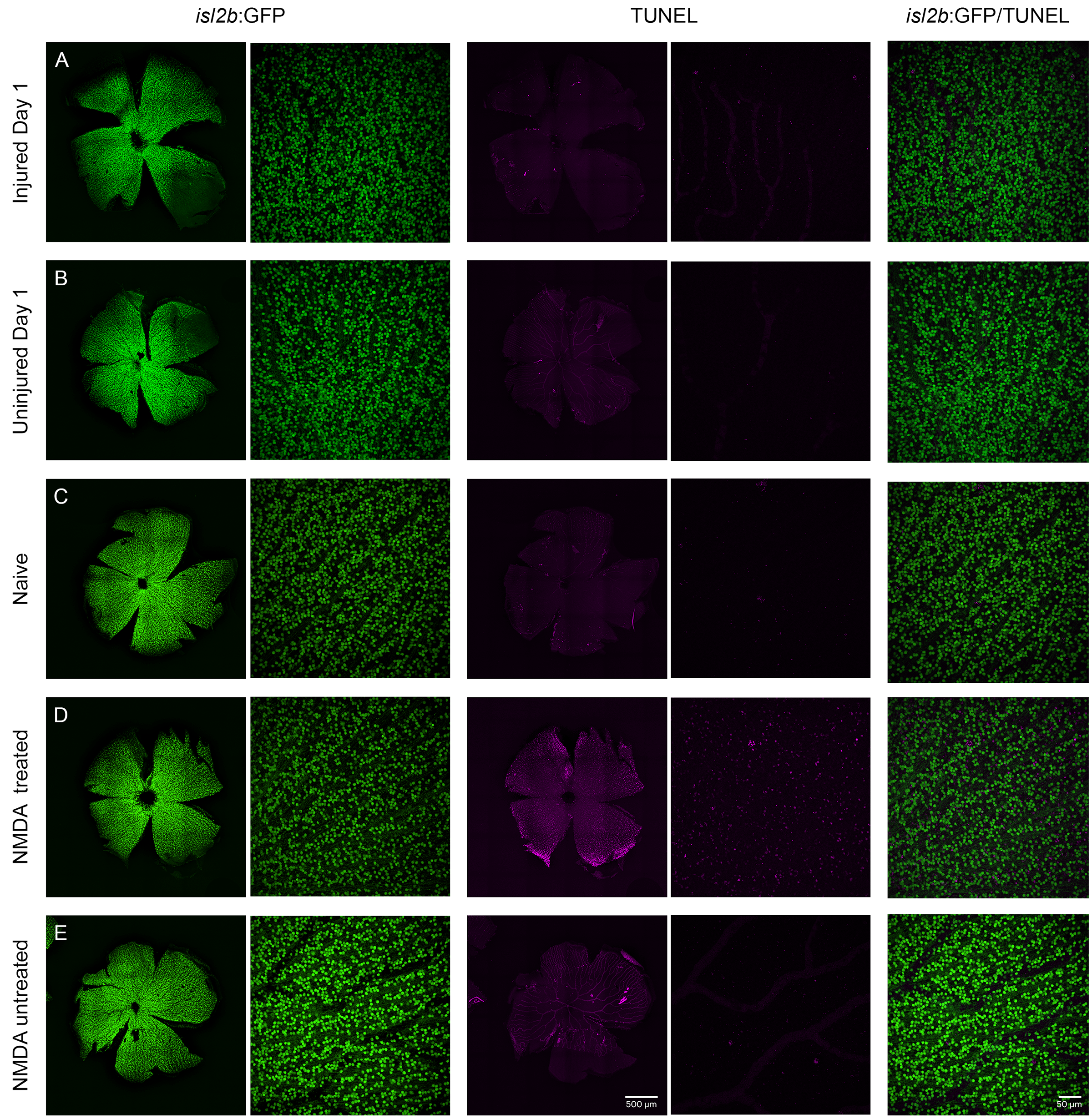

Supplement: S4 Fig — Representative images of high resolution fields of view at 1 day post ONT, naive, and NMDA injured retina (40X objective, n = 24 fields of view per condition). A) Injured retina at 1 dpi do not have TUNEL+ cells. B) Uninjured and C) naive retina also show no TUNEL+ cells. D) NMDA injury elicits substantial TUNEL+ RGCs at 1 dpi compared to E) PBS controls. (TIF) [file pgen.1011879.s008.tif]

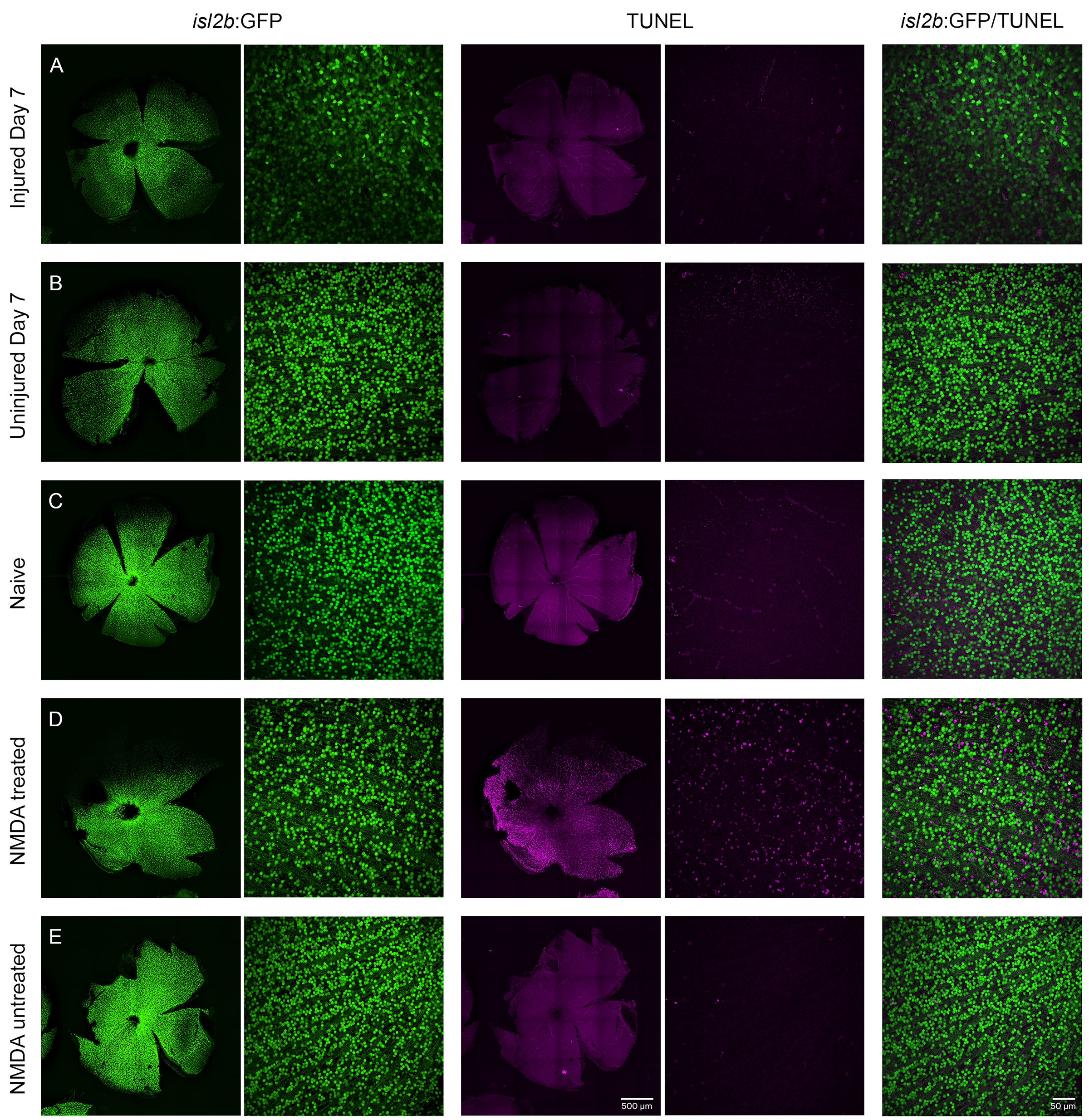

Supplement: S5 Fig — Representative images of high resolution fields of view at 7 day post ONT, naive, and NMDA injured retina (40X objective, n = 24 fields of view per condition). A) Injured retina at 7 dpi do not have TUNEL+ cells. B) Uninjured and C) naive retina also show no TUNEL+ cells. D) NMDA injury elicits substantial TUNEL+ RGCs at 7 dpi compared to E) PBS controls. (TIF) [file pgen.1011879.s009.tif]

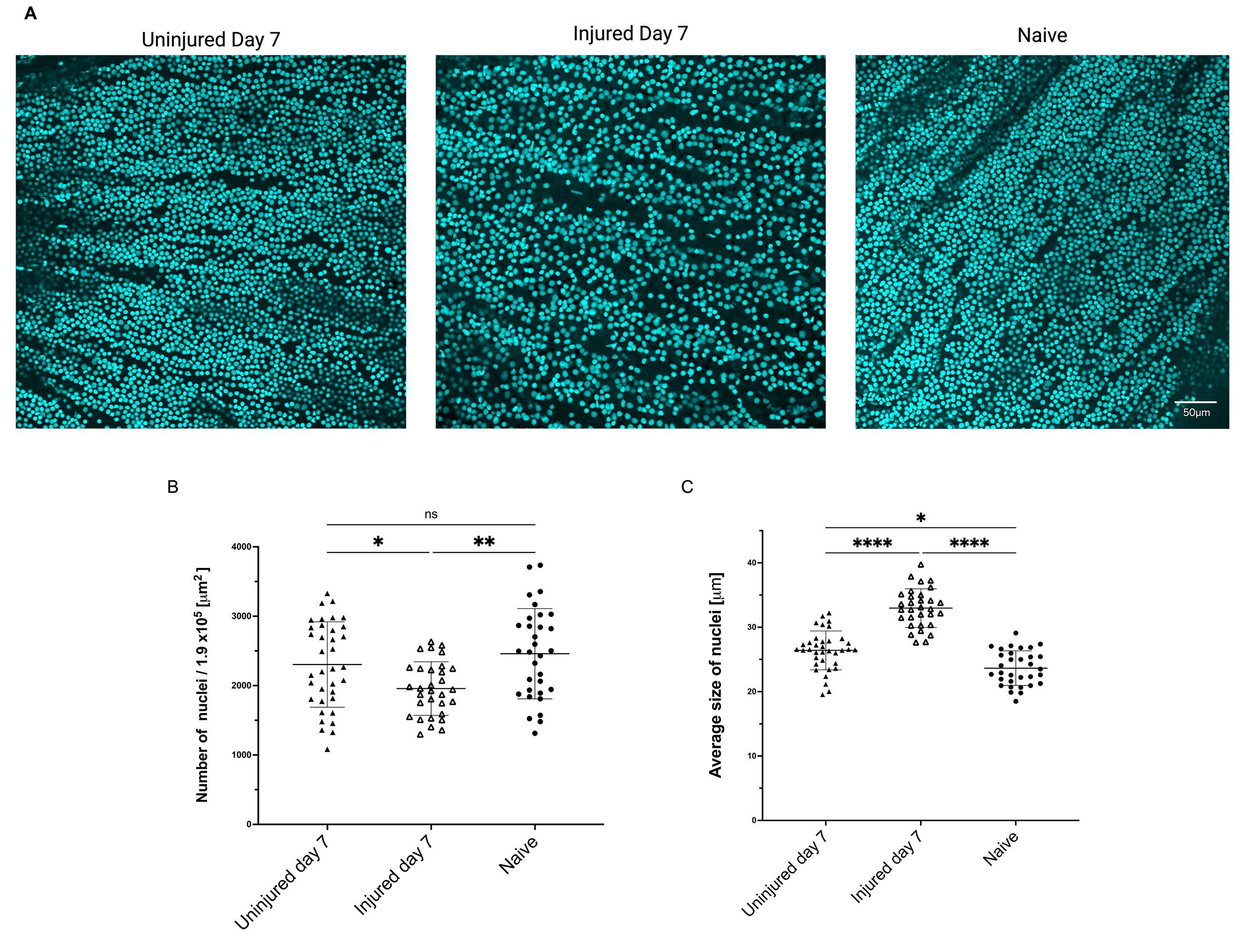

Supplement: S6 Fig — Representative images of high resolution fields of view in 7 days post ONT or naive retinae (40X objective, n = 32 fields of view per condition) show that A) RGC density decreases 7 days post injury compared to naive. B) Injured retinae at 7 dpi have fewer nuclei (M = 1958, SD = 385) compared to uninjured (M = 2304, SD = 616) and naive (M = 2460, SD = 651) retinae. C) Nuclei of injured RGCs at 7 dpi are larger (M = 33, SD = 3) than those in uninjured (M = 26, SD = 3) or naive (M = 24, SD = 2.7) retina. DAPI stains mark nuclei. Scale bar = 50μm. (TIF) [file pgen.1011879.s010.tif]

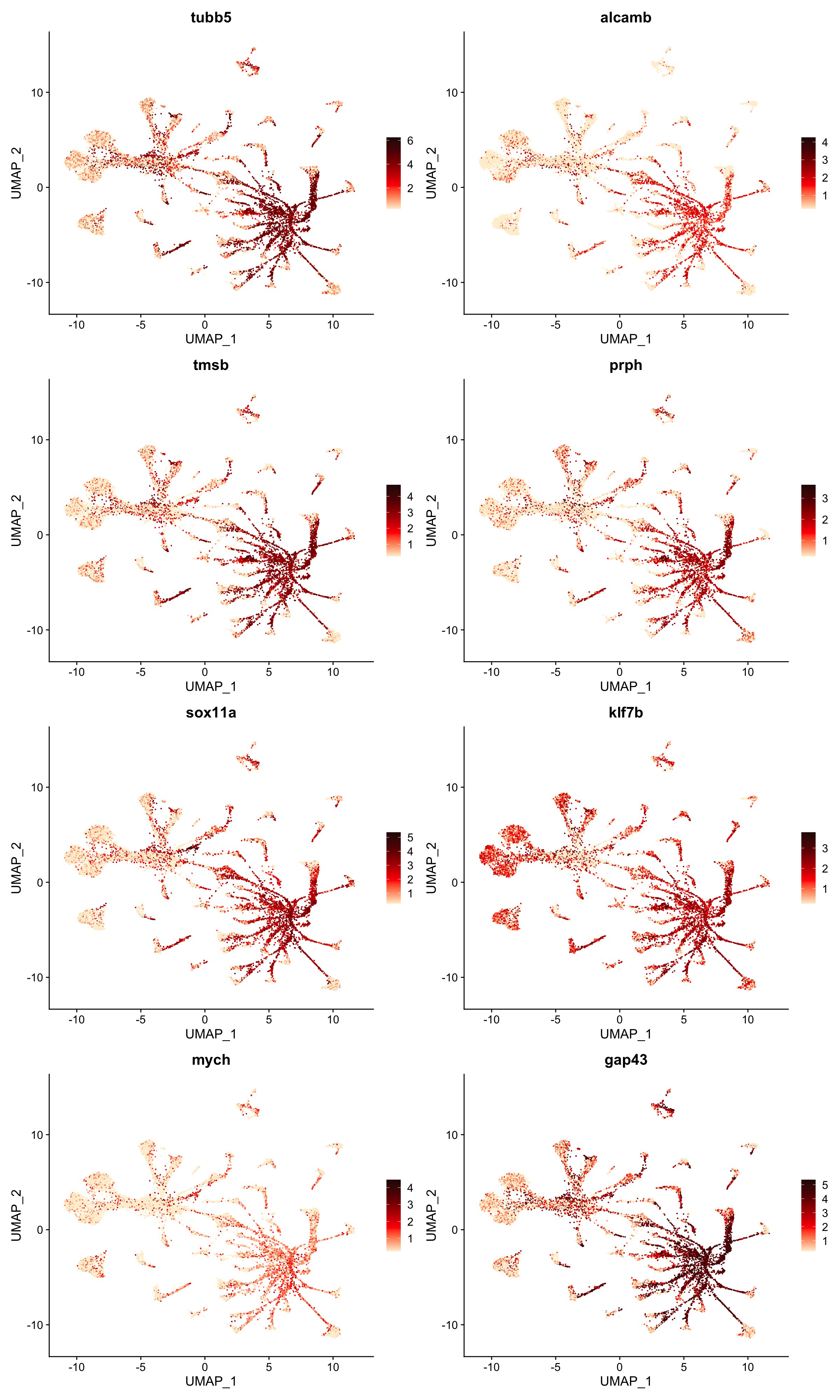

Supplement: S7 Fig — (TIF) [file pgen.1011879.s011.tif]

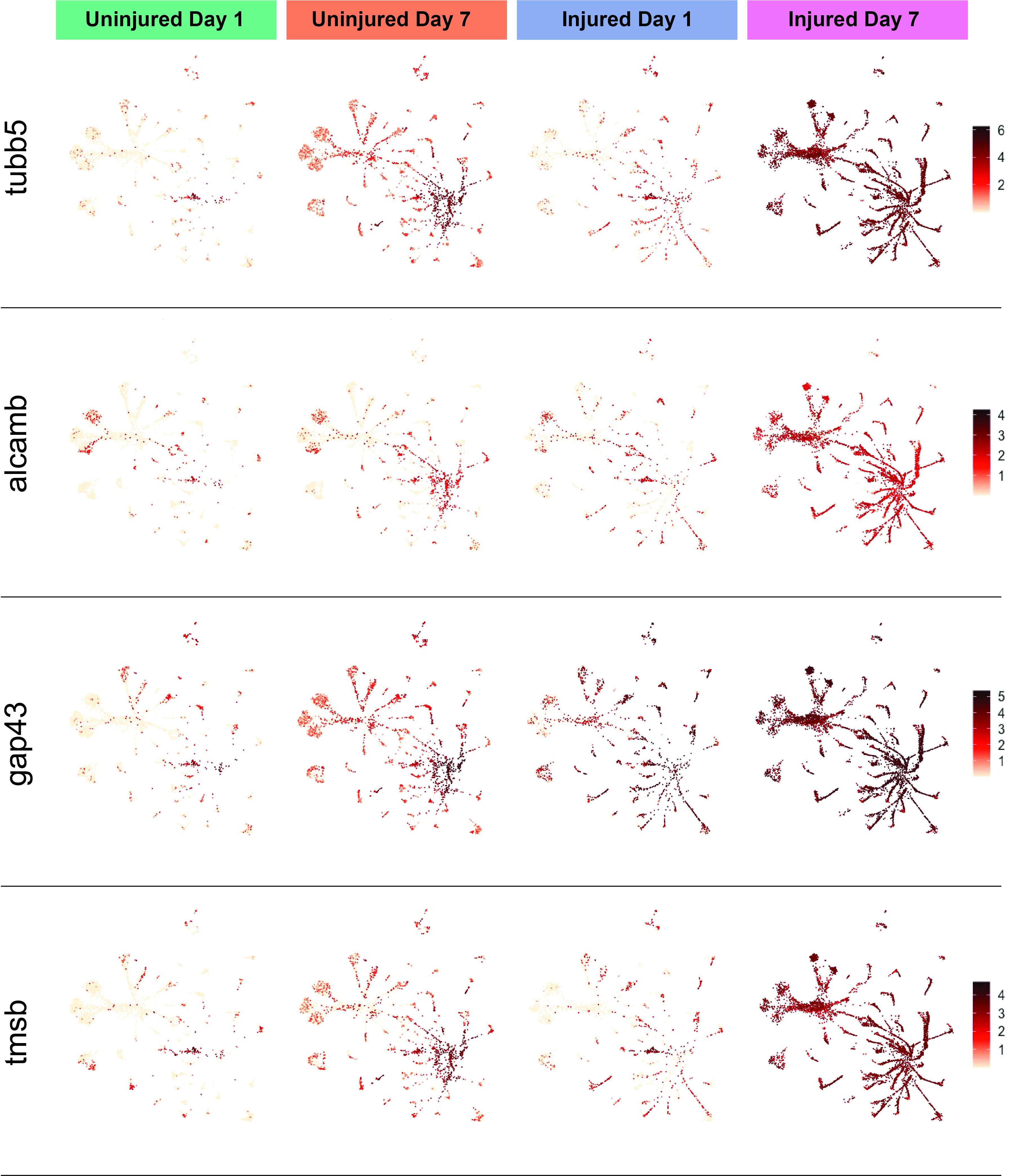

Supplement: S8 Fig — (TIF) [file pgen.1011879.s012.tif]

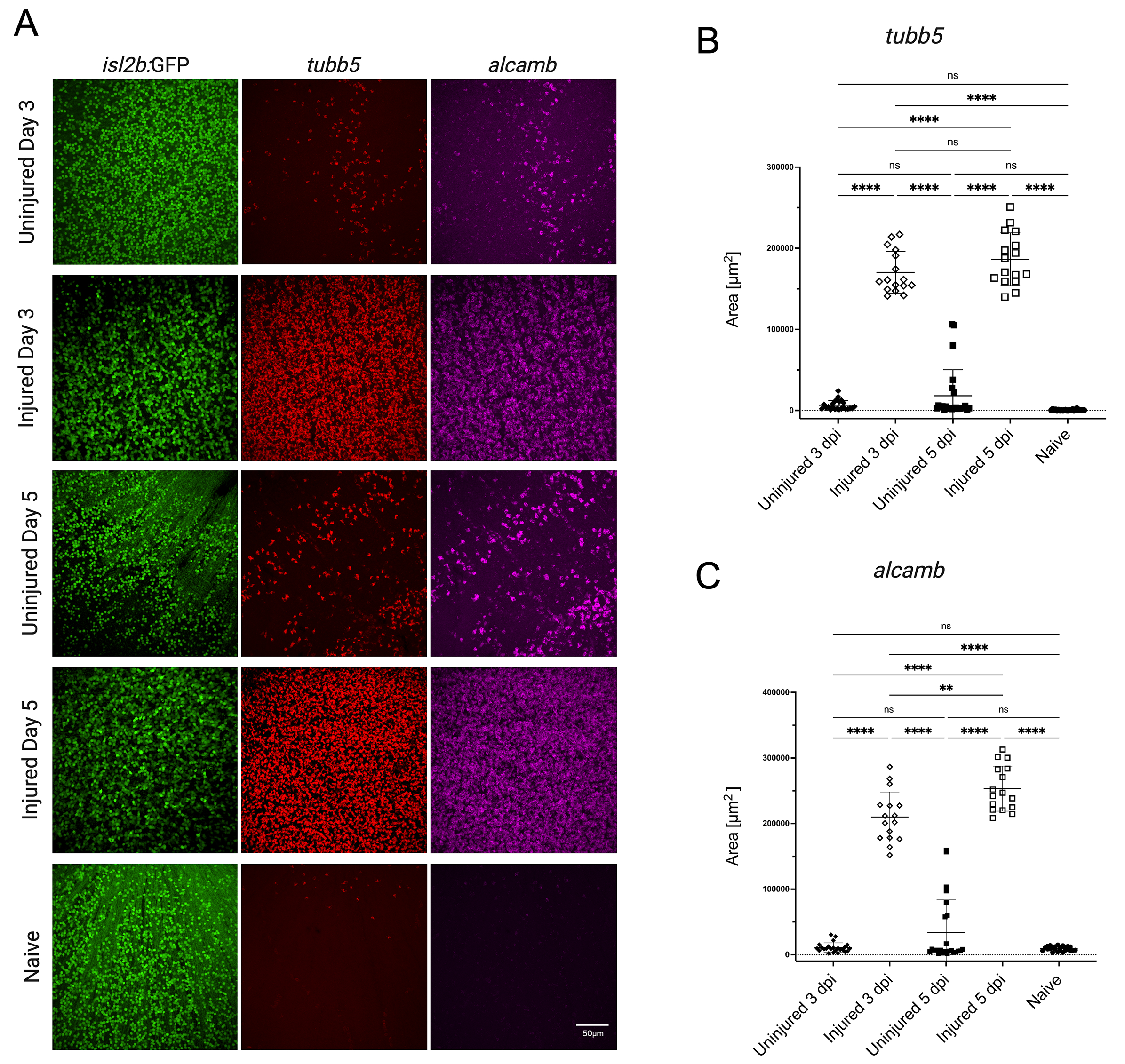

Supplement: S9 Fig — Representative images of high resolution fields of view in 3 and 5 days post ONT or naive retinae (40X objective, n = 16–24 fields of view per condition. n = 3 retina per condition) show that A) tubb5 and alcamb expression in RGCs is robust throughout the retina at both 3 and 5 days post injury B) tubb5 expression is significant as early as 3 dpi compared to uninjured and naive controls and increases significantly at 5 dpi. C) Expression of alcamb is significant as early as 3 dpi compared to uninjured and naive controls and increases significantly at 5 dpi. (TIF) [file pgen.1011879.s013.tif]

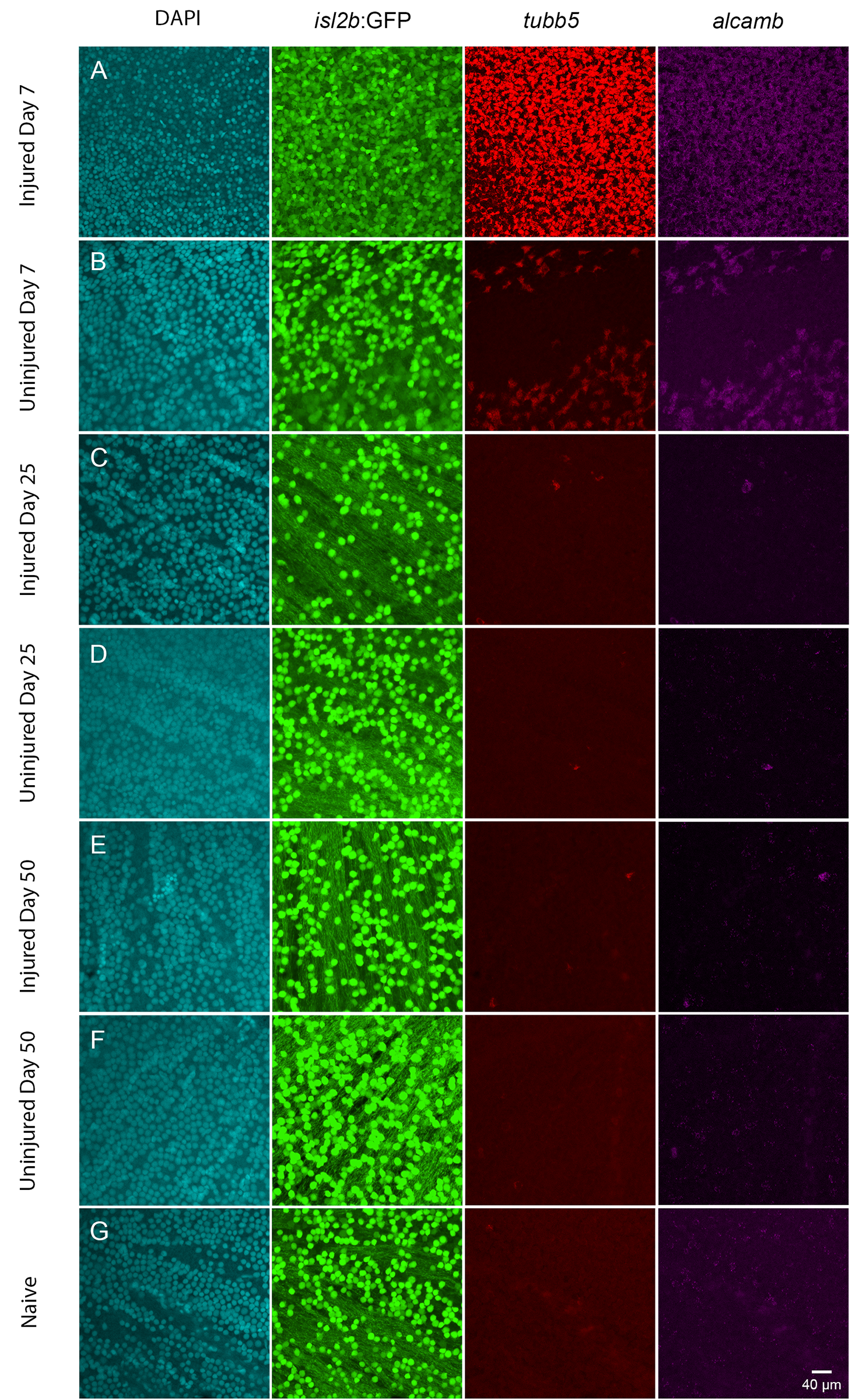

Supplement: S10 Fig — Representative images of high resolution fields of view at 7, 25 and 50 days post ONT or naive retina (60X objective, n = 24 fields of view per condition) show that A) Injured retina at 7 dpi show robust and uniform expression of tubb5 and alcamb. B) Uninjured retina at 7 dpi express tubb5 and alcamb, albeit dispersed. C-F) Expression of tubb5 and alcamb return to baseline levels at 25 and 50 dpi, similar to G) baseline expression in naive retina. (TIF) [file pgen.1011879.s014.tif]
