# Supplementary material for: Zebrafish optic nerve injury results in systemic retinal ganglion cell dedifferentiation
Source: PLoS Genet. 2025 Sep 19;21(9):e1011879. doi: 10.1371/journal.pgen.1011879 (PMC12459811; doi:10.1371/journal.pgen.1011879)
Supplement: Attachment — Submitted filename: Rebuttal.pdf [file pgen.1011879.s016.pdf]

We're grateful to the Reviewers for their time and for the constructive criticisms to improve our manuscript. Many of these required inclusion of additional text or clarification of the original text, so these comments were quite helpful in ensuring that our descriptions of the experiments and data were clear. A few of the comments required additional experiments, which we have also completed and have included these new data in the revised manuscript. Here, we respond to each of the comments, detailing changes made to the manuscript in response.

Included with our resubmission is a manuscript version where these changes have been tracked, as well as a "clean" version where all changes have been incorporated. We hope that with these changes, the manuscript is now acceptable for publication in *PLoS Genetics*. We are confident that the topic and our datasets will be of interest to the broad readership of *PLoS Genetics* and especially to those with an interest in the developmental, regeneration and ophthalmology fields.

Note to reviewers: In revision, we split Figure 2 into two figures and generated multiple new supplemental figures and tables in this updated version of the manuscript. We will be referring to figures/tables according to their *updated figure number* in our responses.

Reviewer's Responses to Questions

Reviewer #1: *The paper by Ali and colleagues characterizes patterns of gene expression in injured retinal ganglion cells (RGCs) in the zebrafish retina. Different scRNA-seq were generated for FACS-enriched isl2:GFP-positive RGCs from control and ON-injured retinas. UMAP-ordering and label transfer for established RGC subtypes revealed treatment-dependent differential distribution among RGC subtypes and k- means neighborhoods. RGC subtype 3 was identified as the least mature cluster of RGCs. Pseudotime and cytoTRACE trajectories originating from cluster 3 were established to identify the distribution of differentiating/maturing RGCs across the UMAP dimensional reduction. Figures 8 and 9 include FISH validation for tubb5 and alcamb in normal and injured retinas with isl2b:GFP RGCs in flat-mounts.*

The paper is entirely descriptive, but contains several interesting observations.

R1 and R3 both indicate that they feel that the manuscript is descriptive. Here, we want to first address this overarching comment. Importantly, we do not disagree with this statement; the paper is descriptive (although with rigorous analyses and validation). However, we feel strongly that being descriptive is not necessarily a disqualifying characteristic of a manuscript, especially in *PLoS Genetics*, where many similar computational studies have been published in the last few years (e.g. [1]). These

published studies have set the stage for many follow-on studies that delve more deeply into distinct aspects of the biological process being examined, and we anticipate that our study will likewise serve as a foundational one for understanding retinal ganglion cell (RGC) survival post-injury.

Indeed, our manuscript is the first to leverage the unique resiliency of RGCs in zebrafish after severe optic nerve injury using state-of-the-art single cell genomic profiling techniques. Understanding the molecular underpinnings of this resiliency holds promise for potentially developing therapies that might save RGCs damaged in diseases like glaucoma or after acute ocular trauma, or minimally, keep them alive longer or slow degeneration. Our computational analyses identified cell dedifferentiation as a novel mechanism underlying this resiliency. We validate our computational results *in vivo*. In addition to this, we also provide a molecular understanding of the contralateral eye response, a facet of the optic nerve injury response that potentially confounds experimental optic nerve injury studies (as well as in other CNS injury paradigms). Our computational experiments are well-designed with biological replicates and our datasets are rigorously analyzed with appropriate statistical measures and conservative interpretations, something not found in many scRNA-Seq analyses being published these days. Our study sets the stage for further analyses in zebrafish as well as studies in mammalian systems where similar biological responses may underlie resiliency. We're excited about these future projects but feel that they are beyond the scope of an initial study and this *PLoS Genetics* manuscript. We are confident that the manuscript, in its current/revised form, rises to the level of expectations for publication in *PLoS Genetics* and will be of broad impact to the field.

Comments for the authors to consider:
- Was there any compelling rational to apply 40 principle components (PCs; dims=40)? Was a JackStraw or Elbow plot used to identify a reasonable cutoff for the number of PC's with high variability?

1. Was there any compelling rational to use IntegratedAnchor and IntegrateData functions to robustly normalize the different libraries? Why not normalize ("aggr" tool) in Cell Ranger or merely used the default Seurat normalization since the libraries were generated, presumbly, with the one Cell Controller run and identical reagents.

   We used IntegratedAnchor because we wanted to integrate our data with Kölsch et. al's (2021) published data to transfer retinal ganglion cell subtype identity, and therefore it was necessary to analyze the data in a similar way. The code was taken directly from Kölsch et. al's published code:

https://github.com/shekharlab/ZebrafishRGC/blob/master/Adult%20Zebrafish%20Clustering.Rmd.

In addition, the libraries were generated in biological triplicate (with Injured Day 1 samples in quadruplicate) and with experiments performed on different days, with large numbers of cells captured per experiment, and therefore library preparation was necessarily performed on different Cell Controller runs. Having experiments performed on different days, as well as looking at subtleties of subtype representation, required controlling for batch effects. This also favored using Seurat integration steps as opposed to the Cell Ranger “aggr” tool of Seurat merge steps. To perform integration steps and label transfer steps, standard Seurat normalization steps are required. When looking at our subclustered subtype 3 cells and when generating our UMAP for injured and uninjured data (Figure 6 and Supplemental Figure 2) we merged the data as opposed to integrating them. We found that biological variance, as opposed to batch effects, was lost when fewer cells were included in the analysis.

2. Was there any filtering for high mitochondrial content to remove dying cells? If so, what was the cut-off?

   We did not filter for high mitochondrial gene content because Kölsch et. al (2021) did not do so. We used their preprocessing methods instead of a pipeline tailored to our data to ensure optimal integration and label transfer with their data. Treating the two datasets the same makes the label transfer more accurate. In addition, we did not find the level of cells highly expressing mitochondrial genes concerning from a quality control perspective. We do not see variation based on experimental state, and we also believe that there are reasonable biological reasons for keeping all cells in the analysis, including potentially stressed cells that may have a high level of mitochondrial expression. We have included our mitochondrial gene representation for each sample in Supplemental Figure 3 and made reference to it in the Methods section (p. 18).

3. Since FindVariableFeature was run with a cut-off at only nfeatures = 1500, it seems unlikely that 40 PCs with a high variability were obtained to justify the approach, unless the average nfeatures per cell was very high. Or, was this lower cuttoff chosen because V2 reagents didn't capture high features/cell?

   We do want to first correct that we originally reported using 1500 features when we actually used 2000 features, which is the suggested number in the Seurat pipeline. This has been corrected in the manuscript.

Similar to our reply found in Point 1 above, the decisions we made regarding the number of features principal components we used were made to keep our preprocessing in line with Kölsch et. al. (2021) such that we could compare datasets and perform label transfer steps.

In addition to allowing the Kölsch et. al. (2021) pipeline guide our preprocessing decisions when deemed appropriate, we also saw little downside to including more principal components in our analysis, other than computational expense.

4. There are no statistics provided for lists of differentially expressed genes (DEGs). It seems necessary to include supplemental data as spreadsheets for the findmarkers output with adjusted p values and logFC for the lists of DEGs for the different comparisons.

   We apologize for this omission. We have included supplemental tables for all differentially expressed gene analyses (Supplemental Tables 1, 3, 4).

5. **Figures:**

   Figure 1: The table/legend with the cluster number and color is not useful and could be removed. It would be helpful to include an occupancy histogram illustrating the percent occupancy for each orig.ident in each UMAP cluster.

   Figure 2 provides an in-depth representation of percent cluster occupancy by each experimental condition (Uninjured Day 1, Uninjured Day 7, Injured Day 1 and Injured Day 7). In this graph, the percent occupancy for each replicate dataset (orig.ident) within each experimental condition (U1, U7, I1, I7) is represented by each individual black mark (dot, triangle, box, or plus). We also acknowledge that Figure 2A was small in the original manuscript so to make this easier for readers, we have separated Figure 2 into two figures in the revised manuscript. New Figure 2 takes former Figure 2A into a larger and easier to view format, which addresses the Reviewer's comment, while the remainder of former Figure 2 constitutes a new Figure 3.

   We also acknowledge the reviewer's comment that the cluster number and color information in Fig. 1C is not useful. However, we feel strongly that this information provides clarity and easy reference, as we are introducing a color vocabulary that is used throughout the paper. Given the limited space these features occupy in the Figure, we'd prefer to keep them.

Figure 1: For consistency, it would be helpful to maintain the same color scheme for the dotplots in panel E as in panel D.

We have made this change in revised Figure 1.

Figure 2: I have no idea what the lines connecting the different k-neighbors in the Milo analysis represent.

As the reviewer notes, Milo analysis computes a k-nearest neighbor graph. The lines are the edges of the graph. Each graph vertex is a single-cell connected by the edges between its neighbors. The graph edges depict the number of cells that are shared between neighborhoods. Milo is described further in the reference noted in the manuscript for readers that are not familiar with this approach [2]. We have also added additional text (p. 6-7) to help readers who are less familiar with Milo analyses.

Figure 3: The feature plots in 3A are difficult to see even with digital zoom. Further these plots might benefit from a min.cutoff and max.cutoff to clean-up and better highlight the highly expressing cells. This comment applies to all other feature plots.

We apologize to the reviewer for not making the goal of Figure 4A more apparent. Our goal here is to emphasize that genes that are well known to be associated with RGC regeneration and cell reprogramming in the literature have high expression in the subtype 3 part of the UMAP. Subtype 3 occupies a large portion of the UMAP (i.e. Fig 1B), so it may appear that the figure needs to be cleaned-up to better highlight expressing cells. However, we specifically used the entire dataset (omitting min and max.cutoff tools) and chose to present the UMAP plots at this size and with this color scheme, to present the data to make this point. This is not intended to be a figure that provides an in-depth examination of the UMAP, but rather emphasizes a singular pattern, centering the expression of these immature RGC genes primarily in the subtype 3 portion of the UMAP. In addition, these specific genes have a relatively binary expression pattern, therefore shifting the max and min cutoff settings are not greatly illuminating. With all of this said, we appreciate that there may be readers that are interested in a presentation of the UMAP data that shows greater complexity and therefore we have (1) included a UMAP highlighting subtype 3 at the top for reference in Figure 4A and (2) We shifted the min.cutoff to q25 for our feature plots, as well as included a larger version as a supplemental figure (Supplemental Figure 7 & 8). In addition, to provide better context for these feature plots relative to other genes, we have included feature plots of all markers for each RGC subtype as a supplemental figure, all using a min.cutoff of q25 (Supplemental Figure 2).

Figure 3: Panel E makes my head hurt – clearly RGC subtype 3 has a distinct transcriptomic profile. The list of genes along the X axis is too expansive and all of the rows of low-expressing RGC subtypes are dead space. These plot could be simplified – perhaps create a new column of metadata for cluster3 vs others to collectively highlight the average expression and percent expression of representative favorite genes. Please choose some of your favorites genes to populate the X axis that perhaps have been previously implicated in important developmental processes. I acknowledge that scRNA-seq provides a deep wealth of data and it is challenging to pick the most relevant and meaningful and favorite genes. It might also help to group and indicate some of the representative favorite genes according to GO category. Again, supplemental data with findmarkers stats would help to further convince readers of the distinct patterns of gene expression. This comment applies to all dotplots.

We understand the reviewer's concern, as there are a lot of data in Figure 4E. We chose to use a dotplot in this way not because we struggled to choose which data were most relevant, but rather because we wanted to represent the *pattern* we found in the data in a holistic manner. The fact that subtype 3 has a distinct transcriptomic profile is not a given without showing a representation of this fact, especially because the narrative of the paper is dependent on RGC subtype 3's unique identity. We understand that dot plots can be (and often are) used to explore and emphasize specific genes. However, our goal for Figure 4E is to highlight the enrichment of a suite of genes that fall within the GO term "Nervous System Development" within cluster 3, rather than emphasize any individual gene constituent of this GO term. All the genes included are statistically upregulated in cluster 3. What we have done is both use this figure to show enrichment of this suite of genes specifically in cluster 3 in a way that can be observed with a quick assessment, but then also included the individual gene names so that if readers do have a favorite Nervous System Development GO term gene, they can look to see if it is included here. This makes the data more accessible to a reader who is unlikely to dig through supplemental files. This figure serves a similar function as a heatmap. However, we prefer the use of a dotplot over a heatmap because it enables the representation of percent expression and average expression within the same figure. We have included the statistical analyses for differential gene expression in supplemental tables as mentioned above (Supplemental Tables 1-4) and added clarifying language in our figure legend.

Figure 4: It would be helpful to include headings for each plot and labels for the X and Y axes in panels B and C. It is not possible to interpret these data without carefully reading the legends and methods, which creates more work for lazy readers (like me).

We appreciate the Reviewer's comment and have revised the Figure accordingly.

Figure 4: the cytoTRACE and pseudotime trajectories are not consistent with respect to clusters 7, 8 and 23. The pseudotime appears to place these clusters earlier in pseudotime, whereas the cytoTRACE places these clusters as more differentiated. It would be helpful to impart some biological commons sense to and ask whether a handful of genes that are well-established to be highly expressed by happy mature RGCs correlate better with pseudotime trajectory endpoints or cytoTRACE more differentiated scores.

We believe the reviewer means that subtype 7, 8, and 23 are considered to be later in pseudotime (more red in Figure 5E) and more differentiated (more yellow in Figure 5A). The reviewer has identified one of the complexities in the dataset, that there is likely more than one process occurring in the data. Ultimately, there is no reason these two analyses should be perfectly correlated, because they do not address the same question. However, the amount of correlation between this independent assessment of dedifferentiation and pseudotime/lineage trajectory suggests that our hypothesis is quite robust.

Pseudotime is about using static data to try to identify the order of a dynamic gene expression associated, in this case, with the endpoint of subtype 3. cytoTRACE 2 is an algorithm that is intended to measure a differentiation state. The assumption by the reviewer is that a cell cannot be late in pseudotime without being dedifferentiated (as defined by cytoTRACE 2). This is not necessarily the case. There are significant complexities to the pseudotime analysis as a result of branched trajectories in the UMAP. Our trajectory analysis suggests that subtype 7, 8 and 23 are at the culmination of separate branching points. Although they share a late pseudotime identity they are not directly connected to subtype 3 in our Monocle3 hypothesis.

An additional caveat is that cytoTRACE 2 analyses are not specific to RGC maturity or zebrafish physiology, but rather a general measurement of maturity and the outcome should be assessed cautiously.

Our interpretation of the difference identified by the reviewer is that RGC subtypes/clusters with trajectories connected to subtype 3 identified in Figure 5D are highlighting subtypes that are actively dedifferentiating into subtype 3 RGCs, while subtype 7, 8, and 23 are not directly dedifferentiating into subtype 3 RGCs. This remains a hypothesis. Ongoing work using lineage tracing, beyond the purview of this manuscript, will test this dedifferentiation hypothesis directly. We have added text to the manuscript to clarify this interpretation.

Figure 6 and 7: It is not clear that the pseudo-bulk analysis is very different than

generating lists of DEGs by findmarkers by orig.ident. If something distinctly different is revealed by pseudo-bulk (which may provide more statistical power), then this should be better highlighted.

We apologize for the lack of clarity in setting up this experiment in the Results. Pseudobulk is a synonym for differential expression analysis when it is performed by grouping samples, such as by orig.ident or by some other type of identity. As the reviewer is aware, the Findmarkers function in Seurat is primarily performed for differential expression analysis across clusters. Pseudobulk is the term that emerged from the use of statistical methods originally built for bulk RNA-seq analysis employed to analyze pooled single-cell data [3]. However, Seurat now has incorporated many of these statistical approaches into their Findmarkers tool, as well as a pseudobulk vignette in their pipeline. Our analysis was not performed using Findmarkers by orig.ident, because this would have found differentially expressed genes in each replicate. Instead, we performed the differential expression analysis combining each experimental condition (Uninjured Day 1, Uninjured Day 7, Injured Day 1, and Injured Day 7). We performed the Wilcoxon rank sum test, as described in the methods. We have added the following additional text to the Results section (p. 9) to ensure clarity about these approaches.

Pseudobulk analyses use single-cell sequencing data to determine differential expression by aggregating cells into larger, biological sample-level observations.

Figure 7: panel C: please include stats for these genes in the violin plots by orig.ident. A supplemental table would be acceptable.

This can be found in Supplemental Table 4.

6. **Discussion:** “specific loci in the contralateral uninjured eye show similar transcriptional responses to that in the injured eye?” I am not sure that “specific loci” is the appropriate term. Perhaps “sets of genes” is better. There was no gene network analysis or chromatin domain interactions inferred from the RNA-seq data.

We have made this edit, thank you.

……………………………………………………………………………………………………

<u>**Reviewer #2:**</u> *In this manuscript, Ali et al. use zebrafish as a model to study retinal ganglion cell (RGC) resilience and regeneration, an important scientific direction. In mammals, including humans, RGCs degenerate and cannot regenerate, leading to irreversible blindness in diseases like glaucoma. Zebrafish RGCs are known to be both resilient and regenerative after optic nerve injury, so they are an appropriate model for*

this study. In mice, scRNA-seq has shown important heterogeneity between RGC subtypes after optic nerve injury; the authors address whether the same is true in zebrafish. Using a combination of RNA-sequencing and bioinformatic methods, the authors identify a putatively regenerative, immature RGC type (Type 3) and show evidence that following injury, there is broad dedifferentiation of RGCs to become similar to Type 3 RGCs. While intriguing, the bioinformatic results are not fully supported by robust in vivo data. Confidence in the in silico data is also hampered by a lack of description of how the methods were performed. These concerns and others are enumerated below.

Figure 1:

Major Point: The UMAP shown in Figure 1B drives much of the later focus on cluster 3. Could the authors please expand on how these samples were merged/integrated and if the subtype assignments are the same as those shown in Supp Figure 1?

We used standard Seurat integration protocols to integrate all of our data into the final UMAP found in Figure 1 to ensure that batch effects did not impact our final analysis. This is described in the methods section on p. 18:

"Briefly, for each sample, filtered matrices were log-normalized using the NormalizeData function with default arguments. Next all genes were scaled and centered using the ScaleData function and highly variable genes were identified using FindVariableFeatures with nfeatures=2000. Samples were then integrated using the FindIntegrationAnchors and IntegrateData functions with dims=1:40. Next, integrated data were re-scaled and PCA, TSNE, and UMAP embeddings were computed using Seurat's RunPCA, RunUMAP, and RunTSNE functions. Lastly, an initial clustering of the integrated data was computed using FindNeighbors (dims=1:40) and FindClusters."

Subtype identity was defined using the seurat label transfer process. This used the Kölsch et al. dataset to identify the same RGC subtypes in our data. This is described in the methods section on p. 18-19:

"Label Transfer and Data Integration
32 distinct subtypes of RGC have been previously identified in zebrafish [25]. Transfer of the subtype labels to our data using these published data as a reference was performed using Seurat's label transfer workflow. Briefly, transfer anchors were identified using FindTransferAnchors and then labels transferred using the TransferData function, both with dims=1:30. Due to technical and biological differences between Injured and Uninjured 7dpi samples and the reference data, we first transferred subtype labels from the reference data to Uninjured 1dpi data and subsequently transferred those subtype labels from Uninjured 1dpi to the remaining samples. We did not identify subtype 32 (the rarest subtype in the reference data) in

our samples. Additionally, cells annotated as subtype 24, 26, and 30 clustered together in our data, so we combined those subtypes into one subtype named 24&26&30. We also identified two novel subtypes, which we annotated as n1 and n2. Our data were also integrated with previously published wildtype RGC adult single cell dataset for analysis [25]. Integration was performed by using the Seurat SelectIntegrationFeatures function, the FindIntegrationAnchors function and the IntegrateData function. UMAPs of subclustered subtype 3 cells and Injured and Uninjured datasets were performed by merging the data."

We did not perform a label transfer from the Kösch et al. data to our UMAP in Supp Figure 1 because we integrated our data with the Kölsch et al. data instead. This is an alternative approach to achieve similar ends. Therefore, the UMAP in Supplemental Figure 1 is independently numbered. However, there is significant correlation. Cluster 3, for example, shows the same marker expression. Ultimately, this difference in numbering is not important to the point of the figure. Here we intend to show that the Uninjured Day 1 dataset provides an adequate control for our investigation, without contending with batch effects that would influence the integrated Kölsch et al. dataset.

We have added the following text to the figure legend to clarify this:

These cluster numbers and colors are not identical to the subtype identities in our final analysis in Figure 1, however, there is significant correlation. Based on marker expression, Subtype 3 is shared in both UMAPs.

The authors mention that they started with >100,000 cells but end up with only ~17,000 RGCs. Were these other ~80,000 cells non-RGCs or were they removed because they were low quality? Please clarify these details about the UMAP and add additional detail about quality control and the excluded cells. Separate UMAPs (non-merged/non-integrated) from injured and uninjured RGCs would also be helpful.

We thank the Reviewer for noting this and that we had not adequately explained it. These ~100K cells passed Cell Ranger's filters and initial quality control filters but were removed because they were part of clusters that expressed marker genes for contaminant cell types. We are unclear as to why so many contaminant cells were isolated, given that our FACs parameters were rather conservative. *isl2b*:GFP has been reported to be expressed in photoreceptors [4], and most of these contaminant cells were photoreceptors, suggesting that low-level GFP expression was detected by FACs. We opted to use the most stringent parameters in our analysis and to ensure only RGCs were included. We have also generated UMAPs of both injured and uninjured data as requested (Supplemental Figure 3). We had language outlining cell removal in the previous version of the manuscript but have now been more explicit on this point with the following language in the Methods section (p. 18):

*“**Single-cell sequencing data processing***

The cellranger count pipeline (version 6.1.2) was used for alignment, producing a filtered matrix containing UMI counts [124]. We used the zebrafish reference genome (Danio rerio.GRCz11.106) and default arguments. Next we used the Seurat package to preprocess and integrate the samples, following the preprocessing workflow outlined by Kölsch et al. (2021) [25] and starting with a total of 104,003 cells [25,125]. Briefly, for each sample, filtered matrices were log-normalized using the NormalizeData function with default arguments. Next all genes were scaled and centered using the ScaleData function and highly variable genes were identified using FindVariableFeatures with nfeatures=2000. Samples were then integrated using the FindIntegrationAnchors and IntegrateData functions with dims=1:40. Next, integrated data were re-scaled and PCA, TSNE, and UMAP embeddings were computed using Seurat's RunPCA, RunUMAP, and RunTSNE functions. Lastly, an initial clustering of the integrated data was computed using FindNeighbors (dims=1:40) and FindClusters.

To focus on RGC populations, clusters that expressed marker genes for contaminant cell types and lacked expression of RGC markers were filtered and removed. Specifically, we removed clusters that had high expression of at least two of the following non-RGC genes: opn1lw2, opn1mw2, opn1mw1, opn1sw2, opn1lw1, gngt2b, gngt2a, rho, crx, pde6c, pde6ga, opn6a, vsx1, glula, cabp5a, cabp2a, gng13b, gad1b, cd82a, gad2, pax6b, pax6a, cd74a, fcer1gl, and apoeb. We also annotated and removed doublets from each sample using scDblFinder [126] with default parameters. Our final dataset consisted of 17,769 cells, with many removed isl2b:GFP^{+} cells likely being photoreceptors [122]. As part of our quality control assessments, we quantified the percent mitochondrial gene expression and assessed batch effects versus biological variation in our analyses (Supplemental Figure 3A-E).”

Minor points: Could the authors please add language justifying the use of their time points for collecting RGCs post-injury? It would be helpful if they were contextualized for unfamiliar readers.

We appreciate this point to provide better context to readers. We have added the following text on p. 5:

“We chose 1dpi as gene expression changes occur rapidly in RGCs after optic nerve injury (e.g [28,29]) while at 7dpi, gene expression changes associated with axon regeneration are robust and axon regeneration is underway [8,30].”

The sentence “it has been shown that specific subtypes are susceptible…” is a bit misleading because it suggests that some mouse RGC types do not die after injury. In Tran et al. it is shown that all RGC types die, but the proportion of cells that die in a given type varies. If the authors agree, they should clarify this point.

We did not articulate accurately in the original manuscript. We have changed the text of the Results section to clarify this point (p. 5).

"The mouse retina progressively loses RGCs in response to optic nerve crush [31]. After the crush injury, RGCs of all subtypes are lost; however, it has been shown that specific RGC subtypes are more resilient to injury where they have higher proportions that survive [26]."

The authors should contextualize the RGC types as much as possible. The cluster numbers are difficult to interpret, so if there is anything known, or that can be gleaned from their data, that would help compare with RGC types in other species, that would be helpful.

We appreciate the Reviewer's request for context and clarification pertaining to the identity of zebrafish RGC subtypes, as we too are curious about the similarity of RGC subtypes across species. Unfortunately, not much is known given that zebrafish RGCs subtypes have only recently been identified computationally in Kolsch et al. (2021) and now confirmed in our study. Comparing these subtypes computationally to other systems where scRNA-Seq data have been generated (e.g. mouse, non-human primates or humans) is not trivial. Likewise, cellular/physiological characterizations have been performed to identify RGC subtypes in zebrafish (e.g. [5,6]), but these cellular behaviors have not yet been mapped back on to the computational/molecular identities. Kolsch et al. 2021 begins to dissect subtype biology by using restricted transcription factors to create transgenic driver lines for some subtypes and then comparing the resulting axon projection patterns to those described previously (e.g. [7] and their response to ON/OFF signals, but that is the extent of work that has been completed, at least to our knowledge. As we note above, we expect that our study and this publication will be foundational, along with Kolsch et al., 2021, to enable some of these future studies. Indeed, as noted elsewhere in this response, one of our immediate follow-up goals is to generate transgenic lines that express fluorescent markers or lineage tracing tools (e.g. Kaede, Cre) under control of subtype 3 regulatory sequences to determine where these are located in the naive retina and to enable tracing of the injury response, with regard to subtype 3 gene expression. These will undoubtedly be exciting studies, if successful, but are beyond the scope of this initial study. Furthermore, we'd also like to do some cross-species computational comparisons of molecular identities of RGC subtypes, using the published mouse, non-human primate and human datasets. This too will be exciting but also take a substantial amount of effort and is beyond the scope of this initial study. With all of this said, we have added some additional text throughout the manuscript to try to provide some additional context to zebrafish RGCs and comparisons that can be drawn to data from other systems.

I cannot find details in the manuscript about when the NMDA injected fish were collected. Please add this detail.

This was included in the previous Methods section, but perhaps not clear as worded. We have modified the text as follows p. 20-21.

"Whole retinae were extracted from ONT fish at day 1 and day 7 post injury and NMDA-injected and control fish at day 1 post injury and processed as described above."

Figure 2:

Major Point: The increase to cluster 3 abundance is the central point of the paper. Could you please elaborate on how many replicates were collected, whether they were biological or technical, and how consistent the replicates were? It is a little bit surprising that the 1 dpi retinas already have a much higher frequency of C3 RGCs than the uninjured 1 dpi retinas.

20 retinas per sample (injured and uninjured at 1 and 7dpi) were dissected and dissociated per experiment and the experiment was repeated three independent times, with the exception of 1dpi which was isolated in quadruplicate. All libraries were sequenced on the same flowcell to eliminate batch effects. Information on replicates was included in the previous manuscript in the Methods section. This information can be found in the revised manuscript on p. 17-18.

As noted in our response to Reviewer 1, we have made Figure 2A larger (new Figure 2), which shows the consistency of data between replicates for each RGC subtype. In addition, we have added a new QC figure which shows occupancy across the UMAP of each biological replicate (new Supplemental Figure 3). These data show that the data are quite consistent across replicates.

We were also surprised by the magnitude of the 1dpi response; however, this is consistent with published literature showing rapid changes in gene expression in RGCs following injury. For example, Sugitani et al., 2023 showed that three of the Yamanaka factors, *oct4*, *sox2* and *klf4*, are expressed in RGCs within hours of optic nerve injury, possibly under control of heat shock factor 1. Our data expand on these studies by showing not only that gene expression changes are occurring rapidly post injury (i.e. by 1dpi), but also by providing single-cell resolution to these changes showing a consistent expression of subtype 3 markers across most RGC subtypes in the injured retina. This likely reflects the robust and rapid regenerative responses displayed by zebrafish after optic nerve injury and the need to dedifferentiate in response to injury as part of the survival and

regenerative responses that underlie repair. Our future goals are to dissect the gene regulatory networks underlying these changes and determine whether they can be manipulated in mammals to enable neuroprotection in injured RGCs.

It is intriguing that isl2b is downregulated and that there is no TUNEL staining detected after optic nerve injury, but would this method be sufficient to detect the ~20% loss of RGCs previously reported? If the authors could show no change to RGC number with an alternative marker this would be more convincing, or they could remove the claim of no RGC loss.

We thank the reviewer for raising this question as it is one that we have been thinking about quite a bit as well. TUNEL is an acute label, in the sense that it detects fragmented DNA, but not the full duration of an apoptotic response, nor does it detect other types of cell death. Our previous data from Chen et al. 2022 utilized a reduction in GFP in RGCs from *isl2b*:GFP transgenics as a readout for cell loss. At this point, we're convinced that this over represents cell loss, as *isl2b*:GFP^{+} RGCs simply downregulate GFP upon injury (in conjunction with the downregulation of many differentiated cell markers, like *isl2b*, as shown in Supplemental Figure 3), and not all RGCs express the *isl2b*:GFP transgene (so our denominator is lower than it should be). In the Chen study, we did detect cleaved caspase-3 staining (~12% of RGCs), so some cells are presumably dying, but not many. Thus, the conundrum with these new data showing limited cell loss through scRNA-Seq and an absence of TUNEL staining.

To address the Reviewers' concern, our own uncertainty, and to be honest/transparent in the manuscript about these results, in revision we counted nuclei in the RGC layer (via DAPI) in naive and 7-day post-injury retinae. These data are included as a new supplemental figure (Supplemental Figure 6). The data show that while there is no reduction in RGC layer nuclei in the Uninjured Day 7 retina, there is a reduction in the Injured Day 7 retina. Confounding this, however, is a recent study noting that there is substantial retinal swelling post-optic nerve transection, and particularly in the ganglion cell layer [8], which would presumably decrease the overall density of RGC nuclei in a flat-mount retinal prep. We previously noted that RGC nuclei also appear larger post-optic nerve injury and quantify that in this new supplemental figure, showing that nucleus size (as inferred by DAPI staining) increases by 39% in 7dpi RGCs. This too could influence counts of RGC numbers, if coupled to increased tissue size.

Putting all of this together, we agree that some RGCs are likely to be dying, but probably not to the degree that we previously thought. We have modified our text in the Results

and Discussion to nuance this point and indicate that while our data support that *most* RGCs survive optic nerve transection, we cannot rule out the possibility that a small fraction die by 7dpi.

We also would like to emphasize here that this is not the main point of the paper (what percentage of RGCs die or survive); rather, our focus is on mechanisms underlying RGC survival post-injury and our data highlight novel mechanisms mediating the survival response in this overwhelming majority population of RGCs that do survive optic nerve transection.

Figure 3:

Major Point: Is this DE analysis being conducted between C3 RGCs and other RGCs across all conditions? If so, would these results contain a mix of C3 baseline properties and injury response programs? Please clarify and consider doing separate DE analysis for injured and uninjured C3 RGCs.

We appreciate the reviewer's question and regret that we were not clear in the presentation of the Results. These questions are addressed in both Figure 3 and Figure 5.

We performed two analyses of C3 RGCs. Our first analysis is found in Figure 4, which is understanding differential expression between C3 (subtype 3) RGCs and all other RGC subtypes. We started by identifying differential gene expression enrichment unique to each subtype or cluster. This DE analysis is an all-against-all comparison. We then show that our C3 cells show DE expression of markers associated with regeneration and immaturity, which has previously been identified as a character of subtype 3 RGCs in Kölsch et al. As the reviewer has noted, this analysis overlooks any injury-response programs over baseline C3 properties. We were also interested in whether these new C3 cells are identical to the endogenous C3 cells found in uninjured eyes or whether they have injury-response signatures. To assess this, we performed our reclustering analysis of C3 cells, found in Figure 6. In Figure 6, we again performed DE analysis but this time we only looked *within* Cluster 3 RGCs to assess differential expression. What we found is that the new C3 cells found at 7 days post-injury are inherently different from the endogenous C3 cells. This can be seen in the occupancy of each cluster found in Figure 6C.

What we ultimately found is that the new C3 cells (clusters A, C, E, F, G, & H) are performing specific functions, primarily immune or regenerative response. We

hypothesize that some cells are actively transitioning from other RGC subtypes, based on sharing some marker gene expression. This analysis performed in Figure 6 is more thorough than DE analysis of injured versus uninjured C3 cells alone. It highlights not only the differences of Injured Day 7 C3 cells from the endogenous C3 cells, but it also exposes the unique Uninjured Day 7 eye response (Cluster D). In addition, this analysis shows that endogenous C3 cells are also found at Injured Day 1 (Cluster B).

We have added the following text to the Results (p.8) to better explain this analysis.

"To better understand RGC dedifferentiation in response to injury, we wanted to more thoroughly analyze subtype 3 cells in our experimental dataset. Subtype 3 RGCs have an immature identity, however, our initial analysis of differentially expressed genes does not investigate the differences between endogenous subtype 3 RGC and the injury response observed. To achieve a greater understanding of this, we first subsetted and re-clustered subtype 3 RGCs (Figure 6A) revealing nine subclusters (Figure 6B, Supplemental Table 3). We assessed differential gene expression found across the subclusters. The outcome of that analysis finds genes that are only enriched relative to other subtype 3 RGCs, not the entire dataset as a whole."

Minor point: Please add a citation for the references to the regeneration and immature markers genes tubb5, alcamb, tmsb, prph, sox11a…

We have added references to these genes in the appropriate sections of the revised manuscript.

Figure 5:

Major Point: Subclustering of C3 seems to reveal four sub clusters that express markers of other subtypes. This suggests that the initial C3 assignment may not be accurate. If these four subclusters are removed, is there still a significant increase in C3 abundance? Can the authors please show individual expression profiles for these clusters to demonstrate more clearly to what extent they express C3 marker genes vs. other subtype marker genes.

When analyzing gene expression within Cluster 3 as shown in Figure 6, the differential gene expression analysis is performed within Cluster 3 only. So for example, Cluster C, found in Figure 5, is enriched with Cluster 9 markers *only relative* to the other Cluster 3 cells, not relative to the data set as a whole. If these cells were enriched overall with Cluster 9 markers relative to the whole dataset, these cells would be assigned a Cluster 9 identity rather than a Cluster 3 identity. That relative relationship is inherent to the way the analysis has been performed. However, the Reviewer does push on a real difficulty within single cell data analysis and biological cell identity, which is that these assignments are somewhat arbitrary. In this dataset, our hypothesis is that cells are

dedifferentiating/transdifferentiating into subtype 3 cells. If this is occurring, we would expect the boundary between subtype 3 cells and the subtype of origin to be non-discrete. We would expect cells to transcriptionally present in an in-between state. What we found is that when we reclustered subtype 3 cells, all injury-response clusters had an obvious functional identity, either in immune response or regeneration, with the exception of the clusters that expressed markers found in other subtypes. We hypothesize that these cells are transitioning identity and have not adopted their new functional identity.

We have edited the following language for clarity (p 8-9):

"The remaining four subclusters share marker expression with other RGC subtype populations (Figure 6F) and are predicted to be close in pseudotime trajectory (Figure 5D,E). These subclusters, occupied by Day 7 cells, have been defined by differential gene expression to be subtype 3 RGCs, but they share marker expression with other subtypes, suggesting that these may be subtype cells that are responding to injury. This response could be a transient transcriptional state as cells dedifferentiate into late-responsive subcluster A cells or as they redifferentiate into other RGC subtypes. Alternatively, these cells may never lose their endogenous subtype marker expression in parallel to displaying a distinct transcriptional response to injury. Taken together, these analyses identify heterogeneity within RGC subtype 3 and, surprisingly, that Uninjured Day 7 RGCs possess a remarkably similar expression profile as Injured Day 7 RGCs. This further supports the presence of a systemic response to optic nerve injury, resulting in a similar dedifferentiation process in RGCs of both the injured and uninjured eye."

The change in Type 3 RGCs in the uninjured day 7 eyes is intriguing. The principal issue with any "contralateral effect" is how the uninjured eye senses the injury to the fellow eye. Previous work in the mouse showed that networked astrocytes redistribute metabolic resources during stress (Cooper et al. 2020, PNAS DOI: 10.1073/pnas.2009425117). The authors should add data showing whether contralateral glia respond in their model or propose another mechanism.

We thank the Reviewer for noting this as we too are intrigued by this response. The Cooper et al., 2020 study is quite interesting in this regard and we incorporated this into the Discussion of the original manuscript. We hypothesize that immune cells mediate the contralateral response, consistent with studies in other systems. We do not have a good astrocyte marker in zebrafish; several have been identified and transgenics made (e.g. *slc1a3b:myrGFP-P2A-H2AmCherry*; [9]) but transgene expression does not perdure into adulthood. We are testing several antibodies and markers to determine efficacy in adults, but as yet, do not have a good marker to assess or manipulate these after optic nerve injury. This is something we hope to address in the future. Text addressing immune cells and the astrocyte study can be found in the Discussion (p. 16):

"It is almost certain that some aspects of the contralateral eye response in zebrafish are mediated by activation of the immune system, as has been observed in other systems. For example, in rat models of unilateral ocular hypertensive glaucoma [116], optic nerve crush [117] or optic nerve transection [118], significant microglia activation is detected in the contralateral eye after injury. Broadly inhibiting microglia activation or inflammation limits contralateral RGC responses [119]. Astrocytes may also be involved in this systemic response to unilateral injury. Astrocytes of the contralateral eye become reactive after unilateral optic nerve crush in mice [120]. Moreover, a recent study showed that astrocytes from a healthy eye can transfer metabolites to the injured eye when it is damaged, but in doing so become more vulnerable to stress and ultimately degenerate [121]. It will be of interest to characterize these responses in zebrafish and leverage the system to better understand the physiological and molecular components of the contralateral eye response."

Without evidence of a mechanism, there is a possibility that these changes are a batch effect of sample collection and processing. Were the seven-day samples collected and sequenced separately from the day one samples? The authors should add clarifying language to the methods and results section.

20 retinas per sample (injured and uninjured at 1 and 7 dpi) were dissected and dissociated per experiment and the experiment was repeated three independent times, with the exception of 1 dpi which was isolated and sequenced in quadruplicate. The Day 7 samples were necessarily collected at a different time from the Day 1 samples given that time was the variable being assessed. The animals were siblings, however. All libraries were sequenced on the same flowcell to eliminate batch effects. We are confident that these changes are "real" and biologically relevant, not simply an artifact of experimental design. As noted above, we have added clarification and additional quality control analyses (Figure 2; Supplemental Figure 3) demonstrating the quality and reproducibility of each replicate. We have made these experimental design points explicit in the manuscript text.

Figure 6:

Minor points: The authors discuss GO terms which is interesting, but it's unclear what the novelty of these findings are and how they relate to models where regeneration does not occur. They authors should change this part to highlight what is novel about their findings and how they are unique to the regenerating retina.

The Reviewer is addressing an interesting question, related to this study and more broadly, across zebrafish regeneration studies: Are zebrafish unique in how they orchestrate these survival and regenerative responses? We think the answer to this is no, in the sense that there is no unique regeneration cassette that enables these abilities,

but rather a variable ability to implement the same transcriptional response. This is supported by work in a number of regenerative systems, including the retina, where many years of excellent work in Muller glia have revealed pathways present in both zebrafish and mammals, but that operate differently between species, enabling one to regenerate (zebrafish) while most of the others (i.e. mammals) cannot. Collectively, these studies have shown us that zebrafish are using conserved genes and pathways for regeneration, but in ways that aren't operating in mammals. The immune system is probably the best example of this dichotomy where pro-regenerative animals have the ability to modulate immune responses in ways that enable regeneration and prevent degeneration and death, while in non-regenerative animals, prolonged immune system activation often leads to further degeneration.

In this study, we identify another such example, where zebrafish RGCs dedifferentiate into a more embryonic state as a result of optic nerve injury, a theme that is starting to emerge in other neuronal systems. Many of the genes and pathways we identified are not unique to fish but are used in zebrafish in a different way than in mammals, which enables these unique survival and regenerative responses. Our text related to the GO terms tries to capture this point by identifying some obvious pathways, showing (anecdotally) examples in mammals where alterations to these genes/pathways/processes affects RGC survival and regeneration, as well as some less obvious pathways (autophagy, endopeptidase activity, proteasomal activity). We agree that these results are not necessarily exciting, in the sense that we have identified just the types of genes that we would expect, but this sort of analysis is necessary for completeness of our story and there are some potentially new areas to study from these data. We are excited by some of these candidates as well as those in other GO categories or amongst the subtype enriched genes, and we've made some changes to the text to highlight a few of these less trodden genes and categories. Importantly, all data are publicly available so anyone in the field can leverage these data to develop their own interesting hypotheses to test candidates and pathways, beyond those that we have outlined in the text.

Figure 7:

Minor points: This GO and KEGG terms that pop out of their analysis lack novelty and don't add to the story of the paper. This figure could be moved to supplementary materials.

We feel strongly that Figure 8 is actually an important analysis in the manuscript as it shows that RGCs in the contralateral eye respond to optic nerve injury by mounting a mirrored response that is molecularly similar to that detected in injured RGCs. This is the novelty of the finding - despite not being injured themselves, molecularly, the response of uninjured contralateral RGCs is the same as RGCs in the injured eye. While contralateral

eye responses have been reported, our study is the first to assess the molecular underpinnings of this process. We feel strongly that this is an important finding and one of interest to the field, given the number of studies using contralateral eyes as controls for injury. The Reviewer's concern, however, highlights that we could better set up the Rationale for this experiment and have added additional text to this point to make clear the importance of the experiment and the novelty of the finding.

Figure 8:

Major point: The variability of signal of the different markers across the retina is concerning. Is there a reason that the staining would look so patchy? The explanation in the text is lacking. The authors say there are "concentric circles of RGCs emanating form the optic disc" but the images provided appear to show a stochastic pattern of staining that is difficult to interpret.
It's not clear what the arrows are pointing to because the entire retinal whole mount is shown. Because this figure presents some of the only biological affirmation of the bioinformatic results, it is important to support the authors' overall conclusions.

We appreciate the Reviewer's comment here and apologize that we did not articulate our data well in the Results/Discussion. We have remade Figure 9 to better highlight this pattern of expression in the Uninjured eye. We clearly observe concentric circles of expression in Uninjured retinae. Our revised Figure 9 just shows the single channel image of the HCR pattern for *tubb5* and *alcamb*, which helps to make these patterns more obvious to the reader. To be clear, *tubb5* and *alcamb* expression are robustly detected in RGCs throughout the entire Injured retinae, when compared to naive retinae, and this is clear from Figures 9,10 and Supplemental Figure 9. In Uninjured retinae, while the pattern itself is consistent between retinae, there is variability in the number and continuity of the concentric circles of expression. Thus, we show two examples in Figure 9. One of these retinae contains at least three continuous rings of expression, emanating outward from the optic nerve head (Fig 9. B1-B3). The second retina contains a single ring with an enriched region of expression in one quadrant of the ring (Fig 9. B1' - B3'). We do not think this expression is stochastic, though mechanistically, we do know how/why the pattern arises. As noted in the Discussion, we hypothesize that the contralateral eye response is the result of immune cell activity. One possibility is that this pattern of expression could somehow reflect the topography of the axons as they bundle in the optic nerve and the mechanism by which (or the degree to which) the injured eye response is conveyed to the contralateral eye. Importantly, in this study we have rigorously characterized the contralateral eye response, showing that it molecularly mirrors the injury response, and have experiments planned to identify the cellular mechanisms underlying it.

Figure 9:

Minor points: It is nice that the authors show uninjured day 7 regions that show variable expression of tubb5 and alcamb. The authors should make a more direct reference to this in the body of the text. In its current form, the manuscript contains very little description of Figure 9 or its relevance.

We have added some additional text in the Results and Discussion related to marker distribution in the uninjured retina. Additionally, as suggested by Reviewer 3, we have also included intermediate time points (3 and 5 dpi) in Supplemental Figure 9.

Reviewer recommendation: Overall, this manuscript provides intriguing data about how zebrafish RGCs survive and assume a more regenerative identity after injury. The manuscript is well written, but some details need to be added and the relevance of some of the bioinformatic results is not clear. Furthermore, the histologic results are shaky and must be improved in order to support the intriguing in silico data. Therefore, I recommend revisions prior to publication.

We appreciate the constructive comments here and that our responses have satisfied the Reviewer, particularly in adding more information about the histological (in situ) data as well as fleshing out aspects of the computational analyses that were unclear in our original submission.

……………………………………………………………………………………………………

Reviewer #3: *The manuscript by Ali et al. characterized the retinal ganglion cell (RGC) injury response following optic nerve transection in adult zebrafish retinas. Using a combination of single-cell RNA sequencing (scRNA-seq) and histological analyses, the authors showed that all RGC types survive injury and adopt a dedifferentiated, immature-like state. Interestingly, the study also shows that RGCs in the contralateral, uninjured eye also exhibit an injury response, albeit limited to specific regions and at a lower level. This work offers new insights into potential molecular targets for promoting RGC survival in optic neuropathies. However, the manuscript remains largely descriptive and would be significantly strengthened by addressing the following points:*

1. The authors reported an increase in the proportion of RGC type 3 following injury and concluded that all zebrafish RGCs survive damage by temporarily shifting into a less mature state, resembling a rare population of immature RGCs found in uninjured animals. This conclusion is not fully supported with current data, given that: 1) Only two timepoints were assessed by scRNA-seq and RNA-FISH; 2) Most other RGC clusters remain clearly distinct from type 3; 3) The conclusion is based primarily on the expression of a few

marker genes (e.g., tubb5, alcamb, tmsb5). An alternative explanation is that injured RGCs share expression of a limited set of genes with type 3, rather than fully transitioning into this state.

We appreciate the Reviewer's concerns and alternative model. Addressing this alternative model first; we do not claim that RGCs are fully transitioning into subtype 3 upon injury. Indeed, we think that this transition is likely partial and transient. Our data identify subclusters of subtype 3 RGCs that share marker expression with other RGC subtype populations and are predicted to be close in pseudotime (Figures 4 and 5). This shared marker expression suggests that these are specific RGC subtype cells that are responding to injury. This response, however, could be a transient transcriptional state as cells dedifferentiate into subtype 3 cells or as they redifferentiate into other RGC subtypes. Alternatively, these cells may never lose their endogenous RGC subtype marker expression in parallel to displaying a distinct transcriptional dedifferentiation response to injury. We do know that expression of subtype 3 markers reverts to a wild-type pattern by 25dpi, indicating that the response is transient, as would be expected upon complete axon regeneration and recovery of visual function post-transection where RGCs would be expected to resume their pre-injured physiological roles. Our current data cannot differentiate between a partial transition model or a full transition and post-recovery but are consistent in showing that there is a transition towards subtype 3 identity. This is a novel and exciting finding as it begins to unravel the unique intrinsic survival and regenerative abilities of zebrafish RGCs and provides several avenues for future study to identify the mechanisms underlying this ability as well as potentially stimulate it in mammalian RGCs that are unable to survive injury.

Addressing the three specific points raised by the Reviewer:

1) The Reviewer is correct in that we only performed scRNA-Seq on two time points, 1 and 7dpi. However, the transition towards subtype 3 identity is clear from these data, with subtype 3 gene expression increasing between these time points. We also performed bulk RNA-Seq at 5dpi and results are consistent with this transition. It is not clear that adding additional scRNA-Seq time points between 1 and 7dpi would reveal more about the transition. It would be of interest to assess recovered RGCs (i.e. at 25dpi) to determine if RGCs return fully to the subtype profiles displayed prior to injury. We did show that subtype 3 gene expression returns to normal at this time, which, when coupled to the known timeframe for the recovery of visual function post optic nerve injury, suggests that they would return to normal. While interesting, we don't feel that this late time point is necessary to support the main conclusion of our current study, which is that RGC dedifferentiation is a component of the injury response in zebrafish.

2) The Reviewer is correct that all RGC subtypes remain distinct in the injured state. However, if our hypothesis is correct, this dedifferentiation is a dynamic process, so without lineage tracing experiments, it is difficult to assess the fate of each RGC subtype throughout this process. What we do see in Figure 6 is that cells identified as Subtype 3 cells, also share markers for other subtypes, suggesting that they are in some type of transitional state. Finally, the primary piece of evidence that led us to a transdifferentiation hypothesis rather than the Reviewer's limited marker expression hypothesis is that there are statistically more subtype 3 cells in the Injured day 7 dataset. This means that cells are sharing expression of more than just a few marker genes, but rather, a whole suite of genes. This is discussed further below. Whether these new cells are *bone fide* subtype 3 cells (endogenous immature RGCs) or are distinct injury-related stem cells that resemble subtype 3 is difficult to parse without significant further investigation. It is possible that only some RGC subtypes dedifferentiate while others express universal markers. We don't find our hypothesis and the reviewer's hypothesis mutually exclusive, however, and future experiments will begin to assess these possibilities.

3) The conclusion that RGCs dedifferentiate to resemble subtype 3 RGCs is based on more than just three markers. While *tubb5*, *alcamb* and *tmsb* are the main genes discussed, given that they were used by Kolsch et al and confirmed in our study to be subtype 3 markers, and two of whose expression we validated *in vivo* post-injury, our scRNA-Seq data identified many other genes that are also enriched in these cells post-injury. For example, using the results of GO analysis, we show that genes associated with regeneration and reprogramming are also enriched. These include: *prph*, *sox11a, klf7b, mych,* and *gap43*, amongst others. Thus, the transition to a less differentiated state upon injury is robustly supported by our computational data and *in vivo* data.

2. To what extent do RGCs adopt the type 3 state after injury? Do all RGC types undergo this transition, or only a subset? Analysis of additional intermediate timepoints would clarify the dynamics of this potential state transition.

Based on our scRNA-Seq analysis, all RGC subtypes shift towards a subtype 3 identity in that they upregulate subtype 3 genes after injury (Figure 1E & 7D). Our Milo and trajectory analyses (Figure 3,5) in addition to our reclustering of cluster 3 (Figure 6A-C) suggests that clusters 9, 11, 12, 19 and 21 have the clearest evidence of support for transition from one subtype to subtype 3 RGCs. Expression of two subtype 3 markers, *tubb5* and *alcamb*, revealed that all RGCs across the injured retina expressed these genes (Figures 9,10, Supplemental Figure 9). To address the Reviewer's concern about intermediate time points, here, we have added additional in situ hybridization analyses for *tubb5* and *alcamb* at 3 and 5dpi. Consistent with our results at 1 and 7dpi, expression in RGCs initiates at 1dpi, is maintained at 3, 5 and 7 dpi and decreases back to baseline

levels by 25dpi. Likewise, the uninjured contralateral eye shows parallel changes in expression. These new data are shown in Supplemental Figure 9. Importantly, these data do not inform us about the transition itself, as the markers are already upregulated at 1dpi. Lineage tracing experiments are necessary to directly assess the transition of a given RGC subtype to a subtype 3 identity. We are working on developing transgenic tools to enable these studies, but this is beyond the scope of this paper. This will also be particularly challenging given that subtype gene expression reflections enriched expression, not exclusive expression, and therefore finding a suitable gene to target is proving to be difficult.

3. It remains unclear whether adoption of the type 3 state actively promotes RGC survival or is merely a by-product of injury. What factors drive RGCs to adopt the type 3 state? Functional testing of candidate regulators would significantly strengthen the manuscript's mechanistic depth.

The predominant response of RGCs to injury is the expression of subtype 3 markers and potentially the transition to subtype 3 RGC identity. As previously reported, subtype 3 RGCs are thought to be an immature RGC population. Our data suggest that zebrafish RGCs of all subtypes dedifferentiate in response to injury, which may promote survival. However, untangling these two potentially interdependent functions is difficult. The field has primarily focused on mechanisms that promote RGC survival in mice and rats after injury, model systems in which nearly all RGCs die progressively after injury without intervention. As noted in the Discussion, mammalian studies have also correlated immature gene expression in response to stress, and possibly contributing to neuronal survival and regeneration in non-regenerative systems (reviewed in [10]). Our data identify dedifferentiation as part of the injury response in zebrafish, which is a novel and exciting finding, and suggest that this may contribute to survival and regeneration, by reverting the RGCs back to a more embryonic state. Disentangling these processes will be difficult and current experiments are underway to understand how subtype 3 genes are regulated upon injury (i.e. what are the upstream factors that trigger their expression upon injury and are these loci poised for expression in adult RGCs). These will be exciting experiments but involve genomic profiling assays (e.g. ATAC-Seq) beyond the scope of this first study to complete. That said, we have also begun to test some candidate genes identified from bulk RNA-Seq, and these include *vim,* which is involved in axon regeneration in other systems [11–13] and *npc2*, encoding a protein involved in autophagy and lipid turnover [14–16]. Mutations of neither resulted in survival or regeneration phenotypes, suggesting that single gene mutations of potential effector proteins may not result in survival and/or regeneration phenotypes and studies of these putative upstream regulators is likely needed. These experiments are underway but will take time to complete.

4. Where are type 3 RGCs located in wild-type retinas? Could they represent immature RGCs continuously generated from the ciliary marginal zone (CMZ)?

This is a question of interest to us as well. To begin to address it, in revision, we performed HCR-based ISH for *tubb5* and *alcamb* in naive eyes, examining the entire retina but also with a focus on the ciliary marginal zone, as this is a site of continued neurogenesis in the adult zebrafish eye. In naive eyes, we do not detect any signal for these markers in the retina and specifically in the CMZ (**Reviewer Figure 1**). Beyond the possibility that these genes are not expressed in the CMZ, the low abundance of subtype 3 RGCs in the naive retina, possibly coupled to the low expression of these markers in the subtype 3 RGCs, may make detecting expressing cells difficult using in situ hybridization. We're working to develop transgenic lines from which we can trace subtype 3 RGCs during development, in homeostatic conditions as well as after injury, but we do not yet have these lines in place and there are challenges to this approach that we have noted above.

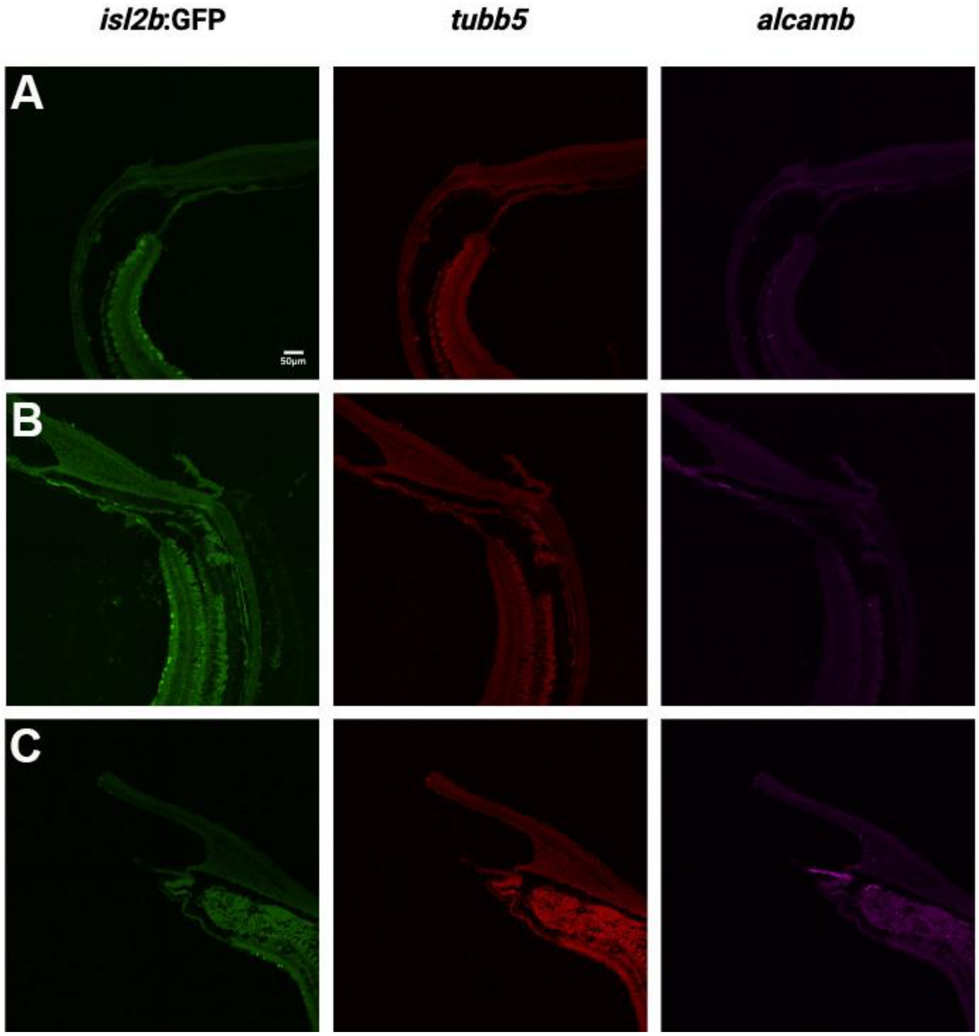

Reviewer Figure 1: *tubb5* and *alcamb* are not expressed in the CMZ of the naive retina. Analysis of CMZ regions from sections of naive retina did not detect expression in the CMZ, despite the presence of newly born RGCs marked by *isl2b*:GFP. A) Ventral CMZ and B, C) dorsal CMZ images taken from retinal sections of 8 month old zebrafish. Scale bar = 50um.

5. RNA-FISH analysis shows an increase in type 3 markers (tubb5, alcamb) from day 1 (low expression) to day 7 (high and widespread expression) post-injury. Including an intermediate time point would improve understanding of expression dynamics.

We thank the reviewer for this comment. To address it, we performed additional HCR-based RNA-FISH labeling for *tubb5* and *alcamb* at 3 and 5 dpi (new Supplemental Figure 9). When combined with our 1 and 7dpi results (Figures 9,10), these new data show that subtype 3 genes are initially expressed at 1dpi, become robustly expressed by 3dpi and are maintained at 5 and 7dpi in the injured eye. Expression returns to baseline by 25dpi (Supplementary Figure 10). Likewise, the uninjured contralateral eye responds similarly with expression detected at 1dpi and maintained through 7dpi.

6. In several plots and heatmaps, the label font size (e.g., cluster numbers, gene names) is too small, making the data difficult to interpret. Increasing the font size would improve readability.

We apologize for this and have increased the font size in several of the figures.

Rebuttal References:

1. Tan J, Sun Y. Alternative promoter usage during organ development. PLoS Genet. 2025;21: e1011635.
2. Dann E, Henderson NC, Teichmann SA, Morgan MD, Marioni JC. Differential abundance testing on single-cell data using k-nearest neighbor graphs. Nat Biotechnol. 2022;40: 245–253.
3. Lun ATL, Marioni JC. Overcoming confounding plate effects in differential expression analyses of single-cell RNA-seq data. Biostatistics. 2017;18: 451–464.
4. Pittman AJ, Law M-Y, Chien C-B. Pathfinding in a large vertebrate axon tract: isotypic interactions guide retinotectal axons at multiple choice points. Development. 2008;135: 2865–2871.
5. Emran F, Rihel J, Adolph AR, Wong KY, Kraves S, Dowling JE. OFF ganglion cells cannot drive the optokinetic reflex in zebrafish. Proc Natl Acad Sci U S A. 2007;104: 19126–19131.
6. Zhou M, Bear J, Roberts PA, Janiak FK, Semmelhack J, Yoshimatsu T, et al. Zebrafish

retinal ganglion cells asymmetrically encode spectral and temporal information across visual space. Curr Biol. 2020;30: 2927–2942.e7.

7. Robles E, Laurell E, Baier H. The retinal projectome reveals brain-area-specific visual representations generated by ganglion cell diversity. Curr Biol. 2014;24: 2085–2096.

8. Shen B-G, Wen Y, Lu S-J, Wei H-Y, Huang S-R, Zhou G-M, et al. From injury to recovery: Spatiotemporal dynamics of the visual pathway during spontaneous structural and functional regeneration after optic nerve transection in zebrafish. Zool Res. 2025;46: 733–749.

9. Chen J, Poskanzer KE, Freeman MR, Monk KR. Live-imaging of astrocyte morphogenesis and function in zebrafish neural circuits. Nat Neurosci. 2020;23: 1297–1306.

10. Traxler L, Lucciola R, Herdy JR, Jones JR, Mertens J, Gage FH. Neural cell state shifts and fate loss in ageing and age-related diseases. Nat Rev Neurol. 2023;19: 434–443.

11. Walder S, Zhang F, Ferretti P. Up-regulation of neural stem cell markers suggests the occurrence of dedifferentiation in regenerating spinal cord. Dev Genes Evol. 2003;213: 625–630.

12. Hui SP, Nag TC, Ghosh S. Characterization of Proliferating Neural Progenitors after Spinal Cord Injury in Adult Zebrafish. PLoS One. 2015;10: e0143595.

13. del Mar Romero-Alemán M, Monzón-Mayor M, Santos E, Yanes CM. Regrowth of transected retinal ganglion cell axons despite persistent astrogliosis in the lizard (Gallotia galloti). J Anat. 2013;223: 22–37.

14. Claudepierre T, Paques M, Simonutti M, Buard I, Sahel J, Maue RA, et al. Lack of Niemann–Pick type C1 induces age-related degeneration in the mouse retina. Molecular and Cellular Neuroscience. 2010;43: 164–176.

15. Wiweger M, Majewski L, Adamek-Urbanska D, Wasilewska I, Kuznicki J. npc2-Deficient Zebrafish Reproduce Neurological and Inflammatory Symptoms of Niemann-Pick Type C Disease. Front Cell Neurosci. 2021;15. Available: https://www.frontiersin.org/articles/10.3389/fncel.2021.647860

16. Tseng W-C, Johnson Escauriza AJ, Tsai-Morris C-H, Feldman B, Dale RK, Wassif CA, et al. The role of Niemann–Pick type C2 in zebrafish embryonic development. Development. 2021;148: dev194258.
